# Assessing parent privacy awareness and coping appraisals in the adoption of child-monitoring technologies

**DOI:** 10.3389/fpsyg.2026.1857799

**Published:** 2026-08-28

**Authors:** Ali Alkhalifah, Umar Ali Bukar

**Affiliations:** 1 Department of Information Technology, College of Computer, Qassim University, Buraidah, Saudi Arabia; 2 Department of Computer Science, Faculty of Computing and Artificial Intelligence, Taraba State University, Jalingo, Nigeria

**Keywords:** artificial neural networks, child monitoring, parental control apps, privacy, protection motivation theory

## Abstract

**Introduction:**

Due to the serious privacy risks inherent in child-monitoring technologies, privacy implications should be a fundamental component of digital parenting guidelines. Several adoption studies indicate that privacy concerns are among the most influential criteria determining users’ intentions toward parental control applications (PCAs). However, the role of PCAs in enhancing children’s digital literacy and cybersecurity awareness remains largely unexamined. Therefore, this study extends protection motivation theory (PMT) to investigate how privacy awareness and coping/threat appraisals influence parents’ behavioral intentions toward PCA adoption.

**Methods:**

To achieve this, we collected empirical survey data from a sample of 541 parents in Saudi Arabia who were recruited via random intercept sampling. A sophisticated, multistage data analysis framework was employed, combining partial least squares structural equation modeling (PLS-SEM), the PLSpredict algorithm, and artificial neural networks (ANNs) to capture both linear and non-linear psychological complexities.

**Results:**

The findings show that privacy concerns significantly influence the behavioral intention to adopt PCAs. The results also indicate that privacy awareness, parental self-efficacy, perceived threat susceptibility, and perceived threat severity have significant effects on parents’ privacy concerns. However, the data do not support the hypothesized effect of assurance mechanisms efficacy (AME). These findings enhance our understanding of user mentality regarding the privacy dimensions of protective parenting software.

## 1 Introduction

Parental control applications (PCAs) collect and manage sensitive child behavior data, including browsing histories, real-time geographical locations, call logs, contacts, and unique device identifiers through specialized technological techniques and methods (Chatterjee et al., 2018). However, as is common in the broader consumer mobile application ecosystem, parental control software frequently integrates third-party advertising and analytic mechanisms to monetize services and aggregate data from user activities, bug reports, and user profiles for decision-making purposes (Future Market Insights, 2026). These background processes expose both parents and minors to severe privacy vulnerabilities, such as unauthorized data profiling and secondary tracking (Fan et al., 2025). To assist parents in navigating these trade-offs and choosing between complex parental control solutions, critical features must be thoroughly evaluated, including functionality, usability, effectiveness, and underlying security safeguards (Bertrandias et al., 2023; Feal et al., 2020).

The critical importance of PCAs is underscored by the global market, which is projected to reach $4.8 billion by 2034, reflecting a desperate parental response to the escalating risks of the digital landscape. Indeed, recent global metrics indicate that approximately 37% of youth now report experiencing cyberbullying, while minors left completely unmonitored face a 40% higher likelihood of exposure to explicit or age-inappropriate content online (Fortune Business Insights, 2026). However, a significant gap remains; despite 92% of parents expressing acute concern over these digital threats, nearly 25% still utilize no technical parental controls or filtering software whatsoever (Boomerang Parental Control, 2026). This striking discrepancy often stems from the exact lack of technical self-efficacy and the creeping privacy anxiety identified in this study, leaving children vulnerable to grooming, data profiling, and unregulated screen habits.

To address these concerns, information systems (IS) researchers have examined security and privacy perceptions across various mobile technologies, including child-monitoring applications (Johnson et al., 2018; Keith et al., 2016). Predictive models demonstrate that perceived security has a beneficial effect on intentional usage drivers (Johnson et al., 2018), while acute privacy concerns actively discourage users from adopting or disclosing information to mobile applications (Keith et al., 2016). Prior studies have established that, while security and privacy are independent conceptual constructs, they remain deeply intertwined and mutually reinforcing (Smith et al., 2011). Consequently, privacy issues in information systems and digital parenting have been studied both independently and in conjunction with security frameworks (Balapour et al., 2020; Mamo and Al-Sarem, 2024).

Despite their relevance, fewer empirical studies have investigated how parental control tools can be structured to safely enhance children’s digital literacy, capabilities, privacy, and cybersecurity awareness (Alrusaini and Beyari, 2022; Gnanasekaran and De Moor, 2025; Mamo and Al-Sarem, 2024; Stoilova et al., 2024; Fan et al., 2025). Scaled compliance audits of the Android parental control application marketplace reveal that these tools are deeply opaque and often fail to meet strict regulatory standards (Feal et al., 2020). Compounding this issue, numerous security breaches targeting parental control solutions have exposed monitored children’s records on a massive scale (Mannan et al., 2020). This compromises children’s online and offline safety, leaving sensitive personal records vulnerable to monetization by malicious actors. Recent empirical reports confirm significant privacy and security risks natively embedded within commercial solutions (Duchaussoy, 2020; Faessler et al., 2017). Nevertheless, parental control tools are viewed as indispensable safeguards by families and educational institutions alike, meaning they are unlikely to be abandoned due to a lack of viable alternatives (Mannan et al., 2020).

Hence, the purpose of this study is to investigate the multi-dimensional factors influencing the adoption of PCAs through the theoretical lens of protection motivation theory (PMT). This constitutes the first empirical study to examine the privacy implications of parental control application adoption from a technological standpoint in the Kingdom of Saudi Arabia (KSA). To capture the complex, non-linear nature of human behavioral intentions, this study utilizes a sophisticated multi-stage analytical framework that combines partial least squares structural equation modeling (PLS-SEM) with artificial neural networks (ANNs).

The remainder of this article is organized as follows: Section 2 reviews the theoretical background and builds the literature context, Section 3 develops the conceptual model and hypotheses, Section 4 details the hybrid research methodology, Section 5 presents the empirical PLS-SEM and ANN findings, Section 6 discusses the findings, Section 7 discusses academic and practical implications, and Section 9 concludes the study.

## 2 Literature review

### 2.1 Parental control applications

To fulfill their monitoring functionalities, parental control applications (PCAs) require highly privileged access to mobile system resources and sensitive data streams. While this deep administrative integration reduces immediate online risks for children, it paradoxically introduces severe data privacy vulnerabilities due to elevated system permissions and unhindered access to privacy-sensitive telemetry (Ali et al., 2020; Alkhalifah and Bukar, 2026).

Empirical mobile ecosystem audits demonstrate that this infrastructure is highly susceptible to exploitation, systemic compliance failures, and hidden surveillance. Reyes et al. (2018) and Feal et al. (2020) uncovered widespread privacy-abusive behaviors, showing that many mainstream PCAs regularly utilize structural tracking libraries and integrate aggressive third-party analytics SDKs. This boundary between domestic protection and unethical monitoring is further highlighted by Maier et al. (2025), whose comparative analysis of parental control systems revealed an alarming prevalence of hidden code execution. Many of these applications deliberately mask their background presence, utilize excessive “dangerous permissions,” and implement intrusive functions—such as remote screenshot capturing, call eavesdropping, and message interception—frequently operating without the user’s explicit knowledge or meaningful consent (Maier et al., 2025).

Despite these acute socio-technical dangers, contemporary literature frequently overemphasizes initial technology uptake while neglecting sustained post-adoption privacy calculations (Jozani et al., 2020). Ultimately, because information privacy is a context-dependent, multidimensional, and dynamic construct, it continuously evolves alongside technological and algorithmic advancements (Acquisti et al., 2015; Mamo and Al-Sarem, 2024; Fan et al., 2025). Examining how parents manage this delicate trade-off between immediate child safety and institutional surveillance is therefore critical to understanding contemporary digital parenting ecosystems.

### 2.2 Privacy concerns

Existing research indicates that privacy governance dictates user attitudes and decisions to adopt mobile applications or disclose personal information. While foundational studies have established that mobile application users’ perceived privacy concerns have a detrimental effect on application permissions (Wottrich et al., 2018; Xu et al., 2009), contemporary research highlights that digital parenting in the post-pandemic era requires a complex “privacy calculus” (Mamo and Al-Sarem, 2024). Recent literature confirms that modern smart-home ecosystems and IoT-driven child-monitoring tools introduce passive data-harvesting risks that actively amplify parental anxiety (Gnanasekaran and De Moor, 2025).

Furthermore, Stoilova et al. (2024) noted a sharp disconnect between family expectations of absolute digital protection and the realities of institutional data tracking. This matches recent evidence that parental enforcement styles vary across cultural contexts; for instance, distinct behavioral shifts and divergent parental roles have been observed in rapidly transforming digital societies (Fan et al., 2025). Current studies therefore insist that static privacy notices are no longer sufficient; future frameworks must develop contingent, dynamic systems where PCA privacy features evolve alongside adolescent behavioral patterns (Mamo and Al-Sarem, 2024). This study addresses this exact gap by utilizing PMT to evaluate how modern threat and coping landscapes alter parental adoption thresholds.

### 2.3 Protection motivation theory

Protection motivation theory (PMT) explains and predicts protective behaviors when individuals confront environmental or technological threats (Rogers, 1975). In information systems (IS), PMT is widely used to evaluate user privacy calculations within mobile software ecosystems (Balapour et al., 2020; Johnston and Warkentin, 2010; Mousavizadeh and Kim, 2015). The framework posits that individuals evaluate risk exposure through two distinct cognitive pathways: Threat appraisal and coping appraisal (Floyd et al., 2000; Maddux and Rogers, 1983). Threat appraisal measures an asset’s perceived vulnerability and severity in relation to external dangers, whereas coping appraisal determines an individual’s self-efficacy alongside the perceived response efficacy of protective mechanisms (Johnston and Warkentin, 2010). While classic PMT assumes that these pathways operate concurrently, modern mobile environments reveal that they often function sequentially, with threat recognition acting as a mandatory prerequisite for coping activation.

In digital parenting, this cognitive evaluation underpins an intricate privacy calculus (Mamo and Al-Sarem, 2024). Parents must constantly balance the high severity and susceptibility of online threats—such as grooming, profiling, and cyberbullying (Gnanasekaran and De Moor, 2025)—against secondary privacy anxiety. Deploying a PCA to mitigate these internet risks requires introducing software into the household that actively monitors a minor’s data telemetry, paradoxically triggering intense perceived privacy concerns regarding developer data governance (Mousavizadeh and Kim, 2015). In addition, PMT accounts for external and interpersonal contextual inputs—such as baseline privacy awareness, breach disclosures, and peer observation—which calibrate these threat metrics (Floyd et al., 2000). Rather than driving immediate technology rejection, elevated awareness heightens parental scrutiny toward systemic safeguards, including encryption and strict data-sharing policies (Stoilova et al., 2024; Fan et al., 2025).

#### 2.3.1 Rationale for adopting PMT over alternative models

To justify model parsimony, PMT was rigorously contrasted against alternative IS behavioral models:

- Privacy calculus theory: It focuses on an economic, rational utility trade-off between immediate risks and explicit benefits. However, it fails to capture the visceral fear, emotional stakes, and protectionist instincts that drive a parent’s desire to shield a child from digital harm (Mamo and Al-Sarem, 2024; Fan et al., 2025).
- Theory of Planned Behavior (TPB): It evaluates generic subjective norms and perceived behavioral controls (Venkatesh et al., 2003). It lacks specialized constructs to model environmental fear appeals triggered by predatory online threats (Johnston and Warkentin, 2010).
- Technology Threat Avoidance Theory (TTAT): It evaluates user avoidance behaviors when defending personal hardware against immediate technical malfunctions. Conversely, PCA adoption represents a proactive, goal-oriented, and assertive protective mechanism deployed on behalf of a vulnerable third party, rather than a passive, self-centric avoidance strategy.

Consequently, PMT provides the most theoretically aligned framework for this study. By explicitly separating threat appraisal (assessing the severity and susceptibility of internet dangers to the child) from coping appraisal (auditing parental self-efficacy and the structural reliability of the application), PMT captures the exact dual cognitive processing parents undergo when adopting child-monitoring technologies.

#### 2.3.2 Exclusion of maladaptive rewards and response costs

To maintain model parsimony and focus specifically on the complex socio-technical privacy paradox, two traditional components of PMT—maladaptive rewards and response costs—were deliberately excluded from the conceptual framework, a scoping practice common in targeted IS security adaptations (Johnston and Warkentin, 2010). In classical PMT, maladaptive rewards represent the perceived intrinsic or extrinsic benefits of maintaining non-protective behaviors. Within the context of digital parenting, the privacy paradox is understood as a multi-determined phenomenon driven by cognitive, contextual, and socio-technical trade-offs rather than purely technological features. While a child might experience convenience or unhindered device freedom from unrestricted internet usage, the target unit of analysis and primary decision-maker in this study is exclusively the parent. Since guardians rarely derive personal rewards, peer validation, or psychological utility from leaving their children exposed to critical cyber threats, maladaptive rewards lack empirical relevance and variance within the parental protectionist calculus.

Furthermore, response costs capture the perceived financial, time, or cognitive penalties associated with executing the recommended protective action. As the vast majority of consumer-facing PCAs are highly automated, free or inexpensive, or embedded natively within mobile operating systems (e.g., Apple Screen Time and Google Family Link), the immediate physical transaction and operational cost of deployment is functionally negligible. Instead, the primary cognitive barrier preventing parents from adopting these tools is an abstract, systemic, and psychological concern regarding corporate data governance, software “piggybacking,” and administrative privacy risks (Mousavizadeh and Kim, 2015). Rather than arising from technological traits alone, this indicates that the paradox is causally driven by a complex mix of bounded rationality and competing motivations. Therefore, omitting response costs allows this study to cleanly isolate how *informational privacy anxieties* alter coping mechanisms without confounding the model with traditional operational or economic constraints (Johnston and Warkentin, 2010).

## 3 Research model and hypothesis development

**Figure 1:**
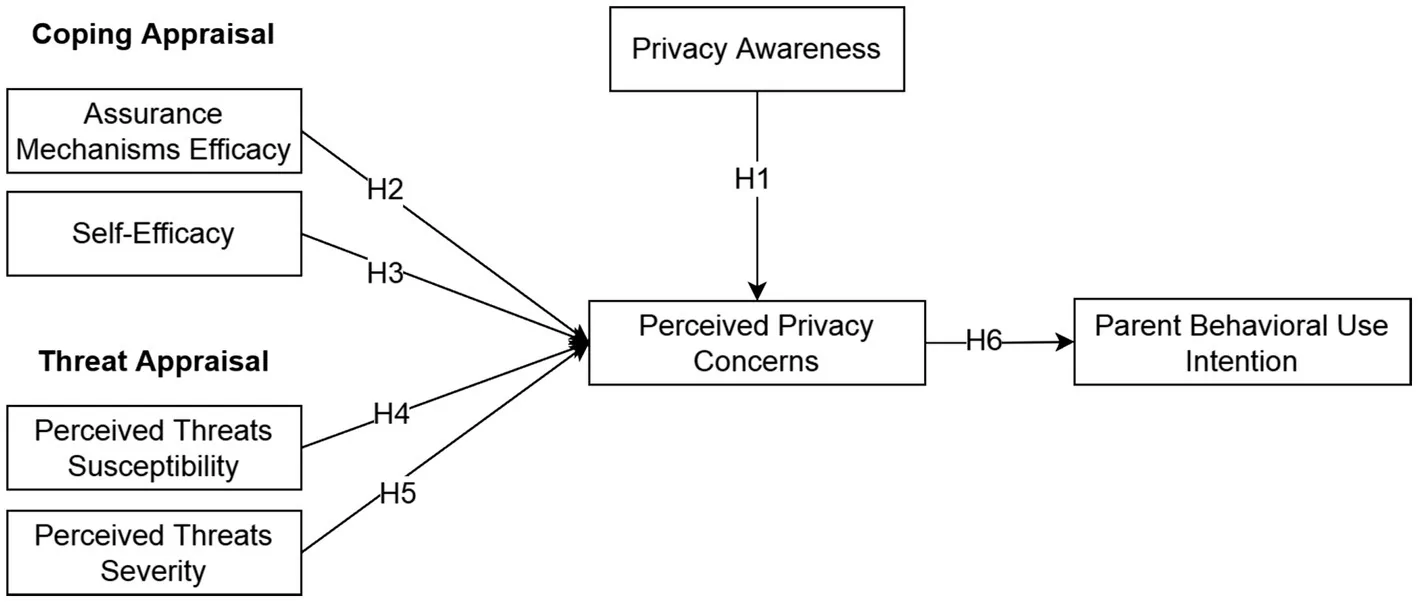
Conceptual model of privacy concerns in parental control application adoption.

Based on PMT, this study assumes that privacy concerns affect parents’ behavioral intentions toward parental control applications through the dual cognitive pathways of threat appraisal and coping appraisal. The conceptual model and hypotheses are presented in Figure 1.

### 3.1 Privacy awareness

Privacy awareness refers to an individual’s general understanding of data privacy procedures and their specific execution within mobile software ecosystems (Malhotra et al., 2004). In the context of technology adoption, privacy awareness dictates that users must be clearly informed regarding when, how, and for what purpose their data are harvested, particularly when information is transferred to third-party entities (Hsu et al., 2020). Crucially, modern mobile application privacy anxieties are not always driven by deliberate developer misconduct; rather, they frequently stem from systemic vulnerabilities, poor coding practices, and the integration of privacy-abusive third-party analytics libraries (Feal et al., 2020). Consequently, policymakers and researchers emphasize the need to enhance user knowledge concerning institutional data governance to counteract the illusion of user control, especially when privacy threats originate from the platform provider itself (Jozani et al., 2020).

Information systems literature establishes that privacy awareness is a powerful, multi-dimensional antecedent that directly shapes users’ privacy behaviors and systemic risk evaluations (Crossler and Bélanger, 2019). Variations in privacy awareness explain why individuals exhibit differing degrees of perceived privacy concerns (PPC) when facing identical technological environments (Balapour et al., 2020). Users who are acutely aware that digital platforms regularly capture, profile, and monetize personal information without explicit consent exhibit significantly higher levels of privacy anxiety than those who remain oblivious to such data-harvesting practices (Smith et al., 2011). As consumers possess varying baseline levels of privacy literacy, their mental models and interpretations of software security fluctuate drastically (Acquisti and Gross, 2006).

Within the domain of child-monitoring technologies, highly aware users are more likely to scrutinize application permissions due to their exposure to reports of unauthorized data disclosures and platform breaches (Balapour et al., 2020). Rather than acting as a passive trait, privacy awareness functions as an active cognitive catalyst that shapes privacy-related perceptions and encourages protective behaviors (Smith et al., 2011). When parents possess an elevated understanding of mobile data collection risks, they naturally evaluate parental control applications with greater structural vigilance, heightening their localized informational anxieties. Hence, we hypothesize the following:

*H1*: Privacy awareness significantly and positively influences parents’ perceived privacy concerns regarding parental control applications.

### 3.2 Coping and threat appraisals

In this study, the “threat” is defined as unauthorized third parties gaining access to the personal information of parental control application (PCA) users (Johnston and Warkentin, 2010; Mousavizadeh and Kim, 2015). Grounded in Protection Motivation Theory (PMT), users process fear appeals and formulate behavioral intentions through two primary cognitive pathways: Coping appraisal and threat appraisal (Maddux and Rogers, 1983; Rogers, 1975). In digital parenting contexts, a parent’s right to data privacy and information control is a necessary prerequisite for application deployment (Feal et al., 2020; Mannan et al., 2020). PMT posits that when individuals believe they possess effective coping strategies, their perceived control over a threat increases, which systematically minimizes their underlying anxiety (Dinev and Hart, 2004).

Coping appraisal is divided into evaluating the effectiveness of the protective strategy itself (response efficacy) and evaluating one’s personal capability to execute that strategy (self-efficacy) (Maddux and Rogers, 1983; Rogers, 1975). In this study, assurance mechanisms efficacy (AME) adapts response efficacy, capturing a parent’s structural belief that technical safeguards—such as end-to-end encryption and transparent privacy policies—act as viable coping mechanisms to prevent data leakage (Bansal et al., 2015; Mousavizadeh and Kim, 2015). Concurrently, self-efficacy (SEF) reflects a parent’s digital literacy and internal assessment of their ability to correctly configure and safely derive benefits from PCAs (Luo et al., 2021; Meng and Qi, 2016). When parents perceive high assurance mechanisms efficiency and high self-efficacy, their perceived control over their personal records improves, which directly mitigates their cognitive friction and localized privacy anxiety (Bowman and Stern, 1995; Mousavizadeh and Kim, 2015). Consequently, an individual’s cumulative coping appraisal dictates their ultimate level of perceived privacy concerns. Hence, we hypothesize the following:

*H2*: Assurance mechanisms efficacy has a significant effect on parents’ perceived privacy concerns.

*H3*: Self-efficacy has a significant effect on parents’ perceived privacy concerns.

Conversely, the threat appraisal process entails evaluating potential vulnerabilities and severe hazards embedded in the transaction between the user and the software environment. When disclosing information through parental control platforms, parents assess both perceived threat susceptibility (TSU)—the likelihood that their family will fall victim to unauthorized access—and perceived threat severity (TSE)—the critical gravity of the negative repercussions if a breach occurs (Mousavizadeh and Kim, 2015). In the information systems literature, this psychological fear and risk calculus regarding data exposure translates directly into localized online privacy concerns (Maier et al., 2025). As parents perceive themselves to be more vulnerable to data exploitation (TSU) or view the impact of information loss as highly severe (TSE), their psychological fear of privacy loss is heavily exacerbated (Mamo and Al-Sarem, 2024; Mousavizadeh and Kim, 2015). Hence, we hypothesize the following:

*H4*: Perceived threat susceptibility has a significant effect on parents’ perceived privacy concerns.

*H5*: Perceived threat severity has a significant effect on parents’ perceived privacy concerns.

### 3.3 Perceived privacy concerns

This study proposes a research model rooted in the dual cognitive mechanisms of Protection Motivation Theory (PMT) to evaluate the adoption thresholds of child-monitoring technologies (Mousavizadeh and Kim, 2015). Perceived privacy concern (PPC) is defined as a user’s systemic belief that an application may mishandle, track, or disclose personal data to unauthorized third parties for improper or unstated purposes (Mamo and Al-Sarem, 2024; Mousavizadeh and Kim, 2015). In online interactions, privacy calculus and behavioral paradigms establish that individuals act strategically to maximize the utility of safe technical deployment while strictly minimizing negative security outcomes (Dinev and Hart, 2006). Consequently, users are fundamentally more willing to disclose personal information and utilize interactive platforms when their localized level of privacy anxiety is secondary to their overall structural confidence in the system (Dinev and Hart, 2006).

In the specific context of digital parenting, exhaustive software audits have repeatedly identified data tracking and administrative surveillance as the most critical concerns shared by both parents and children (Ali et al., 2020; Feal et al., 2020). When processed through the lens of PMT, the fear of privacy exposure directly alters parental attitudes and protective behaviors (Floyd et al., 2000; Mamo and Al-Sarem, 2024). Instead of acting purely as a psychological barrier, heightened privacy concerns systematically dictate a parent’s internal motivation to protect their family’s information infrastructure (Dias and Brito, 2020; Mannan et al., 2020). When parents perceive a severe threat of data leakage or secondary commercial exploitation from the application itself, this detrimental perception creates a behavioral barrier that limits adoption. Therefore, a parent’s calculated privacy anxiety directly determines their psychological willingness to deploy these safety features in their daily lives. Hence, we hypothesize the following:

*H6*: Perceived privacy concerns significantly influence parents’ behavioral intentions to adopt and use parental control applications.

## 4 Methodology

### 4.1 Sampling and data collection

This study gathered empirical data from parents residing in the Kingdom of Saudi Arabia (KSA). Parents represent the primary unit of analysis because parental control applications (PCAs) are explicitly designed to assist guardians in monitoring, managing, and mitigating risks associated with their children’s online behaviors (Alrusaini and Beyari, 2022; Alkhalifah and Bukar, 2026). Driven by protective instincts, parents are highly inclined to adopt innovative mobile tracking solutions despite the latent data privacy vulnerabilities introduced by such platforms. Furthermore, a review of the contemporary information systems literature reveals a pronounced scarcity of empirical research focusing on child-monitoring application adoption among Middle Eastern and broader Asian populations (Alkhalifah and Bukar, 2026; Stoilova et al., 2024; Fan et al., 2025). Consequently, examining this region makes a critical empirical contribution to the global, cross-cultural understanding of digital parenting and localized privacy behaviors.

Data collection utilized a non-probability intercept sampling method across high-traffic shopping malls to capture a diverse cross-section of the target population (Memon et al., 2025; Alkhalifah and Bukar, 2026). Researchers approached parents with minor children at random intervals, balancing intercept times across weekdays and weekends to minimize selection bias. A QR code linked potential participants to the secure digital survey, where they were screened against strict inclusion criteria: Being a legal guardian of a child under 18, holding KSA residency, and possessing familiarity with parental control services.

Data collection was conducted in Arabic, utilizing a rigorous translation and back-translation procedure (English to Arabic and back) to ensure semantic equivalence and cross-cultural instrument validity. Field researchers approached a total of 780 parents; of these, 612 agreed to participate (78.5% initial response rate), yielding N = 541 fully completed and usable responses after data screening. Participants were screened for prior exposure to child-monitoring software and applications, with 42.1% identifying as active current users, 31.8% as past users, and 26.1% as non-users considering adoption.

The study strictly adhered to institutional benchmarks and received formal approval from the Research Ethics Committee at Qassim University. Prior to participation, respondents reviewed a comprehensive data disclosure statement outlining the research objectives, data retention protocols, and confidentiality guarantees. Informed consent was obtained digitally from each participant before data logging commenced, ensuring entirely voluntary and anonymous participation.

### 4.2 Participants’ demographic profile

Following data cleaning and the exclusion of incomplete records, a final usable sample of 541 responses was acquired. This sample size was considered sufficient for conducting structural equation modeling (SEM) and confirmatory factor analysis (CFA), exceeding the minimum thresholds established in the information systems literature (Hair J. F. et al., 2017; Hair J. et al., 2017).

The descriptive analysis of the finalized dataset (*N* = 541) revealed a diverse demographic distribution. In terms of sex, 65.4% of the respondents were female and 34.6% were male. The age distribution demonstrated a mature sample, with approximately 73% of participants being over 30 years old. Family structures were characterized by active parenting demands, as the vast majority of the respondents (69%) reported having more than one child. Furthermore, the sample exhibited a high level of educational attainment, with 63.3% of participants holding at least a bachelor’s degree.

### 4.3 Construct measurement and survey validation

To ensure reliability and validity, survey scales were adapted from pre-validated instruments (Churchill, 1979). Specifically, Privacy Awareness (PRA) was adapted from Malhotra et al. (2004) and Balapour et al. (2020), Assurance Mechanisms Efficacy (AME) from Johnston and Warkentin (2010) and Mousavizadeh and Kim (2015), Self-Efficacy (SEF) from Johnston and Warkentin (2010), and Mousavizadeh and Kim (2015), Perceived Threat Susceptibility (TSU) and Perceived Threat Severity (TSE) from Johnston and Warkentin (2010) and Mousavizadeh and Kim (2015), Perceived Privacy Concerns (PPC) from Mousavizadeh and Kim (2015), and Behavioral Intention (BIN) from Venkatesh et al. (2003).

To ensure proper statistical identification, each construct was operationalized using a minimum of three indicators (Gefen et al., 2011), capturing the variables’ conceptual domains comprehensively, as detailed in Appendix. Items were measured on a five-point Likert scale, ranging from “strongly disagree” (1) to “strongly agree” (5) (Hinkin, 1998). Prior to full-scale deployment, a pilot test using a panel of five IS academics and 10 active PCA users was executed to establish content validity, with adjustments integrated based on their feedback. Items TSU5, TSE4, and PPC4 were deleted during initial confirmatory factor analysis and scale purification due to low factor loadings.

### 4.4 Statistical analysis strategy

The conceptual model was evaluated using a two-stage hybrid analytical approach combining PLS-SEM and ANNs. First, the structural relationships and hypothesized paths were verified using Partial Least Squares Structural Equation Modeling (PLS-SEM) in SmartPLS 4. Path coefficient significance was determined using the non-parametric bootstrapping method with 10,000 resamples to generate robust confidence intervals (Hair J. F. et al., 2017). Beyond standard in-sample model fit indices, out-of-sample predictive performance was rigorously verified utilizing the PLSpredict algorithm (Shmueli et al., 2016).

Second, an ANN analysis was conducted using SPSS v24 to capture potential non-linear relationships and reevaluate the model’s predictive capacity (Chong, 2013). Integrating the learning capability of neural networks effectively optimizes performance and minimizes estimation errors (Alharbi and Alkhalifah, 2024, 2025). Specifically, a multilayer perceptron (MLP) architecture using a feed-forward back-propagation algorithm was implemented to establish the normalized relative importance of the independent predictors relative to the dependent constructs, following established empirical guidelines (Alkhalifah and Bukar, 2026). To prevent overfitting and guarantee generalizability, a tenfold cross-validation technique was applied, partitioning the dataset into 70% for network training and 30% for testing across 10 distinct ANN models.

## 5 Results

Before executing the structural model, the data distribution and variance properties were rigorously evaluated. Common method bias (CMB) was assessed using Harman’s single-factor test (Harman, 1976). Unrotated exploratory factor analysis revealed that a single factor accounted for 41.22% of the total variance, remaining comfortably below the conservative 50% threshold (Podsakoff et al., 2003). This confirms that the dataset was free from significant common method variance complications.

Sampling adequacy and factorability were subsequently analyzed using the Kaiser–Meyer–Olkin (KMO) test and Bartlett’s Test of Sphericity. The KMO test yielded an exceptional value of 0.934, indicating a highly adequate sample for structural modeling. In addition, Bartlett’s test proved highly significant (*p* < 0.001), confirming substantial correlations among the measurement items. While individual indicator distributions exhibited normal deviations typical of behavioral research, the overall multivariate properties and adequacy indicators satisfied the rigorous thresholds required to proceed with variance-based structural equation modeling (Hair J. F. et al., 2017).

### 5.1 Measurement model assessment

The construct reliability of the measures was assessed originally by examining their convergent validity. As illustrated in Table 1, all item loadings exceeded 0.500, ranging between 0.613 and 0.894, indicating that the items and their constructs exhibited an appropriate degree of variation (Chin, 1998; Hair et al., 2019). The items (PRA2, TSU3, and TSU4) with loadings below 0.700 were considered for further analysis because the average variance extracted (AVE) values remained satisfactory (Hair J. F. et al., 2017). The reliability measures for the model’s latent variables are summarized in Table 1. Cronbach’s alpha values ranged from 0.713 to 0.856, exceeding the recommended threshold of 0.70. In addition, the rho_A values exceeded 0.7, ranging between 0.765 and 0.859, thereby demonstrating satisfactory composite dependability. Similarly, the composite reliability (CR) values were greater than 0.80, ranging from 0.806 to 0.912. Furthermore, all AVE values exceeded 0.50, ranging from 0.514 to 0.776 (Chin, 1998; Hair et al., 2019). Surprisingly, these statistical measurement indices indicate that the measurement model fits the data well.

**Table 1:** Item loadings and reliability measures.

Constructs	Items	Loadings	Cronbach’s Alpha	rho_A	Composite Reliability	AVE
Assurance Mechanisms Efficacy	AME1	0.718	0.713	0.734	0.838	0.634
	AME2	0.837				
	AME3	0.827				
Behavioral Intention	BIN1	0.867	0.856	0.859	0.912	0.776
	BIN2	0.894				
	BIN3	0.882				
Perceived Privacy Concerns	PPC1	0.784	0.779	0.801	0.869	0.690
	PPC2	0.861				
	PPC3	0.845				
Privacy Awareness	PRA1	0.841	0.780	0.707	0.806	0.514
	PRA2	0.691				
	PRA3	0.751				
Self-Efficacy	SEF1	0.844	0.782	0.804	0.873	0.697
	SEF2	0.870				
	SEF3	0.831				
Perceived Threats Severity	TSE1	0.879	0.744	0.765	0.807	0.583
	TSE2	0.869				
	TSE3	0.751				
Perceived Threats Susceptibility	TSU1	0.773	0.806	0.812	0.885	0.720
	TSU2	0.828				
	TSU3	0.630				
	TSU4	0.613				

To assess discriminant validity, each indicator’s loading on its assigned construct was compared against its loadings on all other constructs. The results showed that all indicators loaded at or above the loading of the corresponding constructs, as indicated in Table 2. The loadings of each indicator on its construct were considerably higher than the loadings of the indicators on the other variables in the sample. In addition, the discriminant validity of the constructs was assessed using the Fornell–Larcker criterion (Table 3). According to this index, two criteria must be met to establish discriminant validity: (1) the square root of each construct’s AVE is greater than its correlation with other constructs, and (2) each item loads most strongly on the construct with which it is most closely associated (Chin, 1998; Henseler et al., 2015). As shown in Table 3, all values were statistically and considerably higher compared to the linked construct, suggesting that discriminant validity was acceptable.

**Table 2:** Cross-loadings of measurement items.

Construct/Item	AME	BIN	PPC	TSU	TSE	PRA	SEF
AME1	0.718	0.517	0.302	0.455	0.346	0.540	0.519
AME2	0.837	0.430	0.402	0.422	0.479	0.514	0.512
AME3	0.827	0.302	0.432	0.382	0.431	0.428	0.433
BIN1	0.460	0.867	0.309	0.433	0.297	0.544	0.564
BIN2	0.447	0.894	0.266	0.381	0.346	0.552	0.563
BIN3	0.597	0.882	0.297	0.419	0.389	0.587	0.651
PPC1	0.334	0.155	0.784	0.418	0.407	0.413	0.314
PPC2	0.380	0.263	0.861	0.486	0.467	0.517	0.438
PPC3	0.467	0.369	0.845	0.534	0.456	0.506	0.618
TSU1	0.429	0.307	0.513	0.773	0.527	0.494	0.545
TSU2	0.369	0.326	0.440	0.828	0.381	0.429	0.427
TSU3	0.185	0.110	0.331	0.630	0.241	0.222	0.234
TSU4	0.484	0.516	0.355	0.613	0.315	0.508	0.563
TSE1	0.442	0.310	0.498	0.460	0.879	0.618	0.492
TSE2	0.518	0.470	0.465	0.482	0.869	0.625	0.621
TSE3	0.361	0.169	0.365	0.380	0.751	0.441	0.313
PRA1	0.477	0.500	0.502	0.500	0.558	0.841	0.577
PRA2	0.475	0.552	0.340	0.441	0.362	0.691	0.613
PRA3	0.455	0.292	0.468	0.408	0.605	0.751	0.447
SEF1	0.531	0.655	0.449	0.493	0.421	0.605	0.844
SEF2	0.571	0.576	0.529	0.546	0.527	0.614	0.870
SEF3	0.516	0.489	0.469	0.555	0.525	0.566	0.831

The highlighted values represent the primary factor loadings, confirming that each item loads significantly higher on its intended construct than on any opposing construct.

**Table 3:** Fornell–Larcker criterion matrix.

Constructs	AME	BIN	PPC	TSE	TSU	PRA	SEF
Assurance Mechanisms Efficacy	0.796						
Behavioral Intention	0.571	0.881					
Perceived Privacy Concerns	0.482	0.331	0.830				
Perceived Threat Severity	0.519	0.468	0.584	0.717			
Perceived Threat Susceptibility	0.531	0.390	0.536	0.530	0.835		
Privacy Awareness	0.609	0.637	0.581	0.587	0.680	0.764	
Self-Efficacy	0.637	0.674	0.570	0.627	0.581	0.701	0.848

### 5.2 Structural assessment model and hypothesis testing

**Figure 2:**
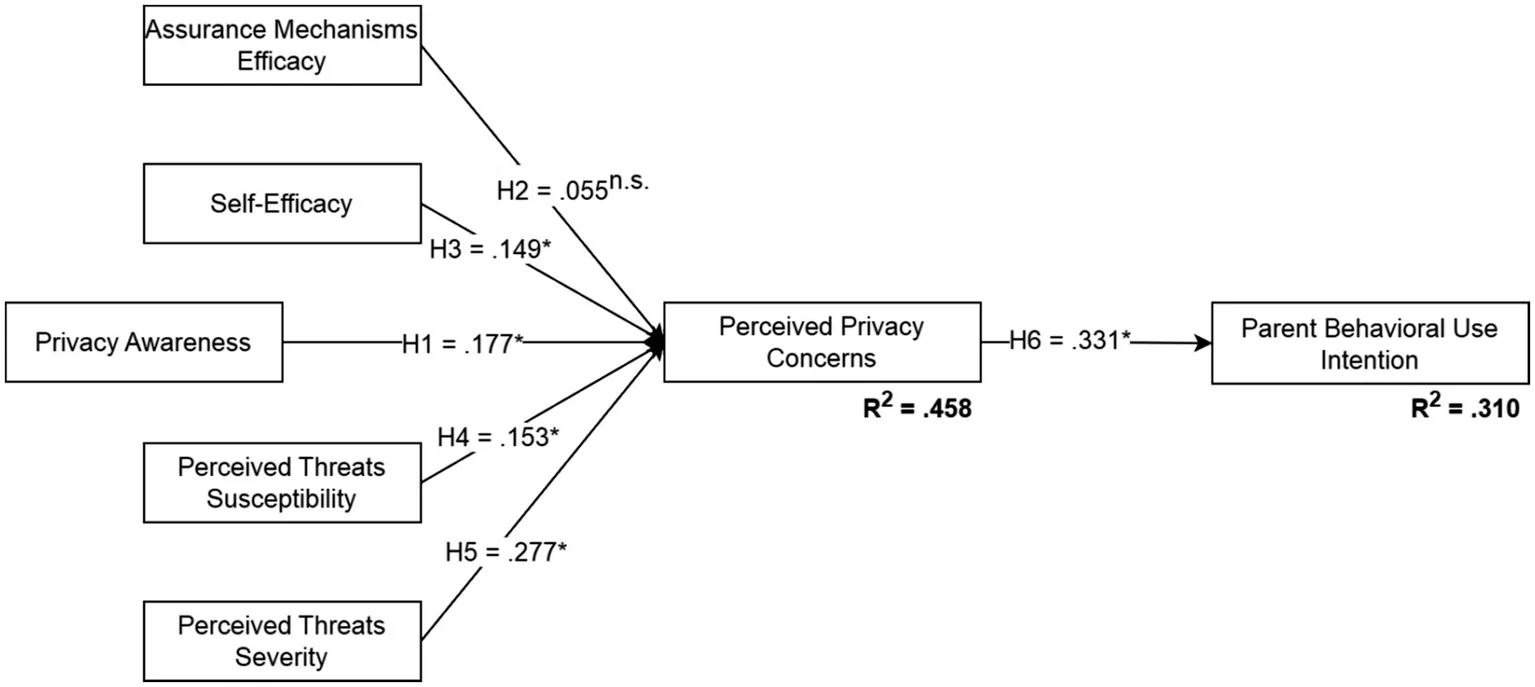
Structural model results. n.s., non-significant; *p < 0.05 = significant.

The findings of the hypothesis testing are illustrated in Figure 2 and discussed in detail in Tables 4–6. Generally, the R^2^ values indicate that the endogenous variables have weak to moderate explanatory power in the model. In most cases, the Q^2^ values are consistent with the R^2^ values (Hair et al., 2019). In terms of predictive relevance, the model demonstrated medium predictive capacity for perceived privacy concerns(Q^2^ = 0.299) and behavioral intention (Q^2^ = 0.281). Similarly, the research model, in particular, was able to account for some variance in respondents’ perceived privacy concerns (R^2^ = 0.458) and behavioral intention (R^2^ = 0.310). Hence, these findings indicate that the structural model explained 45.8% of the variance in privacy concerns and 31% of the variance in behavioral intention.

**Table 4:** Construct PLS-SEM measures.

Endogenous variables	R^2^	Adjusted R^2^	Q^2^	Exogenous variables	Effect size (f^2^)
Perceived Privacy Concerns	0.458	0.453	0.299	Assurance Mechanisms Efficacy	0.003
				Perceived Threat Severity	0.077
				Perceived Threat Susceptibility	0.021
				Privacy Awareness	0.022
				Self-Efficacy	0.016
Behavioral Intention	0.310	0.308	0.281	Perceived Privacy Concerns	0.123

**Table 5:** PLS-SEM structural model results.

Relationships	Β	SD	T values	*p*-values	f^2^	VIF
Privacy Awareness -> Perceived Privacy Concerns	0.177	0.075	2.379	0.017	0.022	2.691
Assurance Mechanisms Efficacy -> Perceived Privacy Concerns	0.055	0.060	0.915	0.360	0.003	1.902
Self-Efficacy -> Perceived Privacy Concerns	0.149	0.058	2.553	0.011	0.016	2.547
Perceived Threat Susceptibility -> Perceived Privacy Concerns	0.153	0.059	2.602	0.009	0.021	2.020
Perceived Threat Severity -> Perceived Privacy Concerns	0.277	0.061	4.533	0.000	0.077	1.842
Perceived Privacy Concerns -> Behavioral Intention	0.331	0.040	8.280	0.000	0.123	1.000

**Table 6:** Summary of hypothesis testing results.

Code	Hypothesis	Supported
H1	Privacy awareness positively influences perceived privacy concerns regarding parental control applications.	Accepted
H2	Assurance mechanisms efficacy has a significant effect on parents’ perceived privacy concerns.	Rejected
H3	Self-efficacy has a significant effect on parents’ perceived privacy concerns	Accepted
H4	Perceived threat susceptibility has a significant effect on parents’ perceived privacy concerns.	Accepted
H5	Perceived threat severity has a significant effect on parents’ perceived privacy concerns.	Accepted
H6	Perceived privacy concerns have a significant effect on behavioral intentions to use parental control applications.	Accepted

The path coefficient statistics reported in Table 6 show that the *f* values (f^2^) were consistent with the *t* values and the hypothesized outcomes. In addition, all variance inflation factor (VIF) values were less than 5, indicating that collinearity posed a small threat to the results (Hair et al., 2019). These findings further support the results of the CMB assessment, KMO test, and Bartlett’s test conducted previously. Hence, the results of the individual hypotheses are discussed below. Specifically, hypothesis 1 was supported, indicating that privacy awareness positively influences perceived privacy concerns regarding PCAs (*t* = 2.379, *p* = 0.017). Hypothesis 2 was rejected, indicating that assurance mechanisms efficacy has no significant effect on parents’ perceived privacy concerns (*t* = 0.915, *p* = 0.360). Next, hypothesis 3 was supported, indicating that self-efficacy has a significant effect on parents’ perceived privacy concerns (*t* = 2.553, *p* = 0.011). Furthermore, hypothesis 4 was supported, indicating that perceived threat susceptibility has a significant effect on parents’ perceived privacy concerns (*t* = 2.602, *p* = 0.009). Similarly, hypothesis 5 was supported, indicating that perceived threat severity has a significant effect on parents’ perceived privacy concerns (*t* = 4.533, *p* = 0.000). Finally, hypothesis 6 was supported, indicating that perceived privacy concerns have a significant effect on behavioral intentions to use PCAs (*t* = 8.280, *p* = 0.000).

### 5.3 PLSpredict

Researchers are encouraged to undertake additional analysis using the PLSpredict method (Shmueli et al., 2016). PLSpredict is a collection of predictive processes that employ PLS path models, along with an evaluation of the predictability of these processes. Therefore, PLSpredict was utilized to ascertain the prediction capacity of the model (Shmueli et al., 2019; Evermann and Tate, 2016). When utilizing the PLSpredict method, it is advised that measurement models meet all applicable criteria and demonstrate an adequate fit. As a result, the reflective measurement models have shown adequate reliability, convergent validity, and discriminant validity (Shmueli et al., 2019; Hair J. F. et al., 2017; Henseler et al., 2015). In response, the PLSpredict technique was implemented, and the predictive value of the model was determined by comparing the root mean square error (RMSE), mean absolute error (MAE), and Q^2^ predict values of the PLS-SEM model with those of the naive benchmark model (LM).

Therefore, prior to considering any other prediction metrics (RMSE and MAE), the Q^2^ predict value is evaluated using the PLSpredict interpretation technique (Shmueli et al., 2019); the PLS-SEM model must outperform the naive benchmark model (Evermann and Tate, 2016). Similarly, the RMSE and MAE prediction metrics were tested due to the non-normal sample structure (asymmetrically distributed data). PLSpredict is performed by executing ten (10) iterations of k-fold cross-validation on the data. Each fold in this example represents a subset of the overall sample, and k represents the number of subgroups in the overall sample. According to the results of the analysis, Q^2^ predict values are less than zero, and both RMSE and MAE values are positive. As a result, the proposed model’s errors are more substantial than those of the linear model. Accordingly, if the PLS-SEM result (compared to the LM) produces lower RMSE (or MAE) values for all the indicators, it is indicated that the model has high predictive capacity. Consequently, the PLS-SEM forecasts have outperformed the linear model (Shmueli et al., 2019). Hence, the findings of this study indicate that the model has weak or insignificant predictive capacity. The results of the PLSpredict algorithm are displayed in Table 7.

**Table 7:** PLSpredict assessment of the original model (PLS-SEM) versus the naïve benchmark model (LM).

Items	PLS-SEM			LM			PLS-LM		
	RMSE	MAE	Q^2^_predict	RMSE	MAE	Q^2^_predict	RMSE	MAE	Q^2^_predict
BIN1	0.992	0.766	0.176	0.839	0.634	0.411	0.153	0.133	−0.235
BIN2	1.341	1.171	0.177	1.022	0.807	0.522	0.319	0.364	−0.345
BIN3	1.257	1.058	0.202	0.787	0.575	0.687	0.470	0.484	−0.485
PPC1	0.726	0.530	0.194	0.715	0.530	0.216	0.010	0.000	−0.022
PPC2	0.654	0.488	0.302	0.656	0.486	0.299	−0.001	0.001	0.003
PPC3	0.593	0.456	0.373	0.581	0.437	0.400	0.013	0.018	−0.027

Behavioral Intention (BIN) and Perceived Privacy Concerns (PPC).

### 5.4 ANN analysis

**Figure 3:**
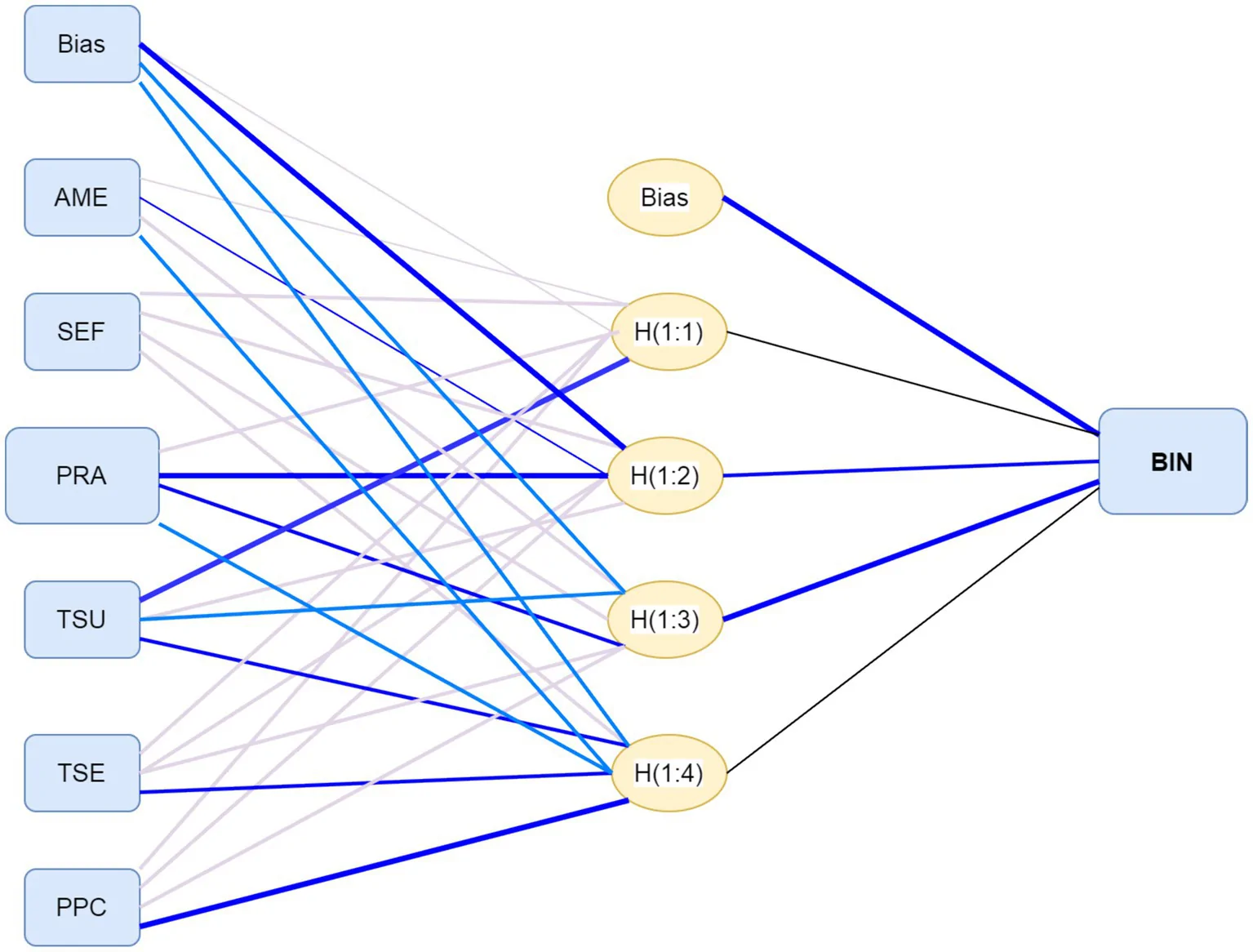
ANN model. Hidden Layer activation function: Hyperbolic tangent; Output layer activation function: Sigmoid; Input neurons: Privacy awareness (PRA), assurance mechanisms efficacy (AME), self-efficacy (SEF), perceived threat susceptibility (TSU), perceived threat severity (TSE), and perceived privacy concerns (PPC).

The ANN method generates a predetermined number of hidden neurons, which are activated using the hyperbolic tangent activation function to achieve the desired outcome. In contrast, the output layer is activated using the sigmoid activation function. Consequently, the RMSE was calculated for each network in the NN model to evaluate the predictive accuracy of the model. In Table 8, it is shown that the ANN model had an average RMSE of 0.397 for the training data and 0.387 for the testing data, indicating that the model’s predictive ability is excellent. A lower RMSE value indicates a more accurate fit and forecast of the data, reflecting a higher level of predictive accuracy. In addition, the number of hidden neurons in the ANN model with non-zero synaptic weights was utilized to determine the importance of external factors. Figure 3 illustrates the ANN model.

**Table 8:** Root mean square error (RMSE) values for the training and testing data.

Network	Input neurons: Privacy awareness (PRA), assurance mechanisms efficacy (AME), self-efficacy (SEF), perceived threat susceptibility (TSU), perceived threat severity (TSE), and perceived privacy concerns (PPC)	
	Training	Testing
1	0.397	0.390
2	0.420	0.370
3	0.385	0.391
4	0.413	0.366
5	0.395	0.403
6	0.410	0.386
7	0.385	0.399
8	0.379	0.406
9	0.390	0.366
10	0.398	0.414
Mean	0.397	0.387
SD	0.014	0.016

SD, Standard deviation.

After determining the expected accuracy and predictive relevance of the ANN model, a sensitivity analysis was conducted to examine the exogenous variables’ predictive potential with respect to the endogenous variables (Ooi and Tan, 2016; Teo et al., 2015; Alharbi and Alkhalifah, 2025). As shown in Table 9, this study calculated the relative significance and the normalized relative value of each exogenous variable. The exogenous variables are presented in Table 9 based on their normalized relative significance and influence on the outcome variable. Interestingly, when the six variables were evaluated using the NN models, privacy awareness was the strongest predictor of privacy concerns in relation to behavioral intention, with a normalized relative value of approximately 100%. Furthermore, the results indicate that assurance mechanisms efficacy (66.70%), self-efficacy (62.24%), perceived privacy concerns (23.38%), perceived threat susceptibility (14.55%), and perceived threat severity (9.27%) were significant predictors of behavioral intention. Among these variables, assurance mechanisms efficacy and self-efficacy were the most influential predictors. Consequently, privacy awareness was the strongest predictor of privacy concerns in relation to behavioral intentions to adopt PCAs, whereas perceived threat susceptibility and perceived threat severity were the weakest predictors.

**Table 9:** Sensitivity analysis.

Network	Input neurons: Privacy awareness (PRA), assurance mechanisms efficacy (AME), self-efficacy (SEF), perceived threat susceptibility (TSU), perceived threat severity (TSE), and perceived privacy concerns (PPC)					
	PRA	AME	SEF	PPC	TSU	TSE
1	0.372	0.256	0.165	0.093	0.075	0.038
2	0.358	0.283	0.209	0.071	0.069	0.009
3	0.318	0.229	0.255	0.114	0.043	0.041
4	0.378	0.191	0.220	0.060	0.074	0.076
5	0.367	0.202	0.254	0.090	0.065	0.022
6	0.324	0.246	0.268	0.124	0.035	0.004
7	0.352	0.271	0.206	0.087	0.057	0.027
8	0.395	0.243	0.212	0.057	0.044	0.049
9	0.380	0.250	0.222	0.058	0.032	0.058
10	0.396	0.245	0.233	0.078	0.032	0.017
Mean (AVG)	0.364	0.2416	0.2244	0.0832	0.0526	0.0341
Normalized RI % (AVG)	100	66.70	62.24	23.38	14.55	9.27

RI, Relative importance.

## 6 Discussion

This study developed an extended Protection Motivation Theory (PMT) framework to evaluate the socio-technical drivers of Parental Control Application (PCA) adoption among parents in Saudi Arabia. While PCAs are functionally essential for shielding minors from online hazards, contemporary software audits reveal that these applications often require highly intrusive permissions and embed hidden tracking, analytics, and advertising libraries that expose families to secondary data exploitation (Feal et al., 2020; Maier et al., 2025; Fan et al., 2025). The empirical findings from our hybrid PLS-SEM and ANN analysis provide critical insights into how parents evaluate these competing digital threats and coping mechanisms.

### 6.1 Evaluation of the threat and coping appraisal pathways

A primary objective of this research was to determine how localized threat and coping appraisals shape parents’ PPC. Regarding the threat appraisal pathway, the PLS-SEM results indicate that both perceived threat susceptibility (H4: *t* = 2.602, *p* = 0.009) and perceived threat severity (H5: *t* = 4.533, *p* = 0.000) exert a significant positive influence on PPC. This reveals that parental privacy anxiety is deeply sensitive to the perceived likelihood and critical gravity of data mishandling or unauthorized access by third-party elements within the application infrastructure (Mousavizadeh and Kim, 2015). When parents perceive that digital vulnerabilities are both severe and highly probable, their internal risk calculation intensifies, escalating their baseline privacy concerns (Mamo and Al-Sarem, 2024).

Conversely, the coping appraisal pathway revealed a striking divergence between internal parental capabilities and external technical trust. Parental self-efficacy (SEF) emerged as a significant determinant of privacy concerns (H3: *t* = 2.553, *p* = 0.011). This reinforces the notion that a parent’s internal digital literacy and confidence in safely configuring monitoring tools directly alter their psychological comfort when deploying tracking systems (Luo et al., 2021; Meng and Qi, 2016).

Crucially, however, hypothesis H2 was rejected, as the linear path coefficient for Assurance Mechanisms Efficacy (AME) showed no significant effect on parents’ perceived privacy concerns (*t* = 0.915, *p* = 0.360). In linear modeling, this suggests that standard structural guarantees—such as static privacy policies, regulatory seals, or listed encryption disclosures—fail to directly mitigate or alter a parent’s immediate privacy anxiety. This lack of linear significance may stem from generalized parental skepticism toward developers’ declarations or from the inherent inability of non-technical users to manually audit the backend behavior of mobile software (Ali et al., 2020; Feal et al., 2020).

### 6.2 The role of privacy awareness in privacy concerns

Our findings strongly support the hypothesized influence of privacy awareness (H1: *t* = 2.379, *p* = 0.017), confirming it as a critical positive antecedent of PPC. This finding aligns with prior information systems literature, which suggests that variations in privacy literacy explain why consumers exhibit highly divergent risk perceptions within identical software environments (Balapour et al., 2020; Smith et al., 2011). Parents who are actively informed about institutional data-harvesting practices, permission piggybacking, and mobile security vulnerabilities exhibit significantly higher structural vigilance. Rather than acting as a passive trait, elevated privacy awareness serves as an active cognitive catalyst that dismantles any superficial “illusion of control,” driving parents to critically evaluate the underlying data protection metrics of child-monitoring platforms (Jozani et al., 2020).

### 6.3 Impact of privacy concerns on behavioral intention

Ultimately, the structural model validated that PPC exert an exceptionally strong, significant effect on parents’ BIN to adopt and use PCAs (H6: *t* = 8.280, *p* = 0.000). This commanding *t*-statistic highlights that privacy considerations are not merely secondary thoughts; rather, they form the primary behavioral gateway determining whether or not parents will execute their protection motivation via software implementation (Dinev and Hart, 2006). When parents realize that the application used to protect their children simultaneously threatens family data sovereignty, a severe adoption barrier is triggered (Alkhalifah and Bukar, 2026).

### 6.4 Synthesis of hybrid predictive analytics

To ensure analytical rigor and address the reviewers’ requests regarding predictive validation, this study contrasted out-of-sample PLS-SEM forecasting with an ANN sensitivity analysis. While the linear PLSpredict algorithm demonstrated relatively modest out-of-sample predictive power for the structural model, the non-linear ANN architecture yielded an exceptionally low RMSE value, validating the model’s high predictive accuracy when accounting for non-linear human decision-making processes (Chong, 2013). The structural and predictive variations between these two analytical techniques are explained in Table 10.

**Table 10:** Methodological comparison of PLS-SEM and ANN sensitivity analysis results.

Predictor construct	PLS-SEM results	ANN sensitivity rank	Rationale for findings
Privacy Awareness (PRA)	Supported (*t* = 2.379)	1st (Most Effective)	Consistently identified as the primary determinant of parental risk assessment across both modeling techniques.
Assurance Mechanisms Efficacy (AME)	Rejected (*t* = 0.915)	2nd	The ANN analysis captured critical non-linear interactions, where AME becomes highly operational only when combined with high digital literacy.
Self-Efficacy (SEF)	Supported (*t* = 2.553)	3rd	Confirms that internal user capability heavily drives the cognitive processing of technology safety.
Perceived Privacy Concerns (PPC)	Supported (*t* = 8.280 to BIN)	4th	Functions as the vital psychological pivot linking appraisal pathways to behavioral intention.
Threat Susceptibility (TSU)	Supported (*t* = 2.602)	5th	Validates that vulnerability perceptions are critical but secondary to internal awareness.
Threat Severity (TSE)	Supported (*t* = 4.533)	6th	Validates that threat impacts are processed less intensely than internal/system capabilities.

While the ANN sensitivity analysis captures complex, non-linear contributions across predictors, the relative importance metrics provide complementary rankings of predictive contribution rather than serving as formal tests of statistical hypotheses. Specifically, relative importance values do not establish statistical significance (*p*-values), directionality, explicit moderating interaction terms, or rigid threshold cutoffs. Instead, the ANN findings complement the linear PLS-SEM path model by highlighting that variables such as Assurance Mechanisms Efficacy (AME) exert a strong non-linear influence on predictive accuracy when evaluated within feed-forward architectures.

The notable discrepancy regarding AME—which was non-significant in the PLS-SEM path analysis but emerged as the second most influential predictor in the ANN sensitivity analysis—highlights a critical methodological and theoretical contribution. As symmetric linear modeling (PLS-SEM) assumes purely additive, linear relationships, it often fails to capture complex, multilayered cognitive patterns. The deep-learning capability of the feed-forward back-propagation ANN successfully unmasked a non-linear relationship, demonstrating that AME does not operate in an isolated or purely additive manner.

Instead, institutional assurances and technical safeguards (e.g., encryption protocols) interact with user perceptions in a non-linear fashion, becoming highly meaningful once a parent’s baseline privacy awareness and self-efficacy reach specific thresholds (Alharbi and Alkhalifah, 2025). Thus, while a simple increase in generic assurances does not linearly drive behavioral intentions, the neural network proves that assurances remain a vital component of the parental coping mechanism when combined with high privacy awareness.

## 7 Research implications

This study makes several distinct contributions to the academic discourse on IS and digital governance. While consumer privacy has been extensively investigated, this research represents one of the first empirical attempts to model how privacy-specific threat and coping appraisal pathways concurrently dictate the adoption dynamics of parental control applications (PCAs). By extending Protection Motivation Theory (PMT) to include latent technical risk variables, this study successfully shifts the academic dialog away from traditional, purely economic utility trade-offs (e.g., standard privacy calculus) toward a deeper understanding of localized, protection-driven risk calculation.

Furthermore, by utilizing a hybrid PLS-SEM and ANN approach, this study addresses calls in behavioral IS literature to move beyond purely symmetric, linear models. Unmasking the non-linear significance of structural safeguards (AME) proves that classical models often obscure critical user dynamics, thereby providing a more methodologically rigorous blueprint for future cross-cultural investigations into family-tracking technologies.

From a practical standpoint, the empirical findings offer actionable strategies for mobile software developers, platform administrators, and digital literacy advocates:

- Strategic Re-engineering of Privacy Policies: Currently, application developers treat privacy disclosures as compliance mechanisms designed to satisfy minimum industry guidelines. However, this study demonstrates that elevated parental privacy awareness directly alters adoption thresholds. Developers should transform standard privacy policies into proactive, interactive educational tools. By utilizing clear and accessible disclosures that explicitly explain *how* data transmission occurs and how families can mitigate background profiling, manufacturers can build sustainable brand equity while alleviating deep-seated parental concerns.
- Contextualizing Privacy Guidance within Software Interfaces: Given that parents frequently overlook routine data-exposure vectors—such as allowing minors to download unvetted secondary gaming applications that demand excessive permissions—developers should integrate contextual security prompts. Privacy control dashboards within PCAs could feature automated password-strength auditors, simplified data-sharing toggles, and live notifications regarding common third-party SDK tracking tactics.
- Targeted Public Awareness Campaigns: As baseline privacy literacy is a dominant predictor of risk calculation, public institutions and child advocacy groups must invest resources in structured digital safety campaigns. Educating guardians about the systemic risks of permissions hijacking and unauthorized telemetry transfers will empower them to make highly informed and secure software choices, ultimately raising baseline privacy standards across the family-oriented software market.

## 8 Limitations and future directions

Although this study provides valuable empirical and methodological insights by integrating PMT, PLS-SEM, and ANNs to assess child-monitoring technology adoption, several inherent limitations must be acknowledged. First, the cross-sectional design captures psychological perceptions at a single point in time, limiting the ability to draw strict temporal or causal inferences regarding how parental privacy concerns evolve over time. Second, the model evaluates self-reported behavioral intention rather than objective system usage logs, leaving room for an intention–behavior gap. Third, the reliance on non-probability intercept sampling in urban shopping hubs may introduce localized selection bias and restrict generalizability to rural demographics. Finally, as the study was conducted within a single-country context (Saudi Arabia), caution should be exercised when generalizing these findings to different regional, regulatory, or socio-cultural environments. Future research should employ multi-wave longitudinal designs, objective usage metrics, and broader cross-cultural samples to validate and extend these findings.

## 9 Conclusion

This study provides the first multistage analysis examining the roles of privacy awareness and coping and threat appraisals in shaping perceived privacy concerns and behavioral intentions to adopt PCAs. The findings initiate a discussion on privacy awareness, coping appraisal, and threat appraisal as antecedents of privacy concern. Of the six hypotheses examined, the results support five and reject one. Consequently, does the promise of PCAs to protect children outweigh the risks associated with their data collection and processing? Therefore, this study hopes to enhance parental and regulatory awareness of the privacy risks posed by some of these applications. Similarly, the study emphasizes the importance of supplementing current benchmarking initiatives with privacy analysis to assist developers in creating better applications and to help parents select the best application while taking these factors into account.

## Data Availability

The raw data supporting the conclusions of this article will be made available by the authors, without undue reservation.

## Appendix

### Research items

**Table:**

Constructs	Code	Items	References
Privacy Awareness	PRA1	Companies seeking personal information online should disclose the way the data are collected, processed, and used.	Malhotra et al. (2004), Balapour et al. (2020)
	PRA2	A good parental control app privacy policy should have a clear and conspicuous disclosure.	
	PRA3	It is very important to me that I am aware and knowledgeable about how my personal information will be used.	
Assurance Mechanisms Efficacy	AME1	When parental control app uses privacy assurance mechanisms, my information are more likely to be protected.	Johnston and Warkentin (2010) and Mousavizadeh and Kim (2015)
	AME2	I believe that the privacy assurance mechanisms that parental control app uses help me to keep my information private.	
	AME3	I think the privacy assurance mechanisms that parental control app uses are effective.	
Self-Efficacy	SEF1	It is easy for me to use privacy assurance mechanisms on parental control app.	Johnston and Warkentin (2010), Mousavizadeh and Kim (2015)
	SEF2	It is convenient for me to use privacy assurance mechanisms.	
	SEF3	I am able to use privacy assurance mechanisms without much effort.	
Perceived Threats Susceptibility	TSU1	My information is at risk for being released to unauthorized people.	Johnston and Warkentin (2010), Mousavizadeh and Kim (2015)
	TSU2	It is likely that my information will become available to unauthorized people.	
	TSU3	It is possible that my Information will become available to unauthorized people.	
	TSU4	It is likely that others get access to my information without my permission.	
	TSU5*	It is probable that others get access to my information without my permission.	
Perceived Threats Severity	TSE1	If my information released to unauthorized people, it would be very bad for me.	Johnston and Warkentin (2010) and Mousavizadeh and Kim (2015)
	TSE2	If my information released to unauthorized people, it would be a serious danger.	
	TSE3	If my information released to unauthorized people, it would be significant danger.	
	TSE4*	If my information be available to unauthorized users, it would be risky.	
Perceived Privacy Concerns	PPC1	I am concerned that parental control app is collecting too much information from me.	Mousavizadeh and Kim (2015)
	PPC2	I am concerned that parental control app will use my information for other purposes.	
	PPC3	I am concerned that parental control app will share my information with other parties.	
	PPC4*	I am concerned that parental control app does not protect privacy of my information.	
Behavioral Intention	BIN1	I likely to use parental control app to monitor my child now or in the future.	Venkatesh et al. (2003)
	BIN2	I will try to use parental control app to assist me in monitoring my child in my daily life.	
	BIN3	I intend to continue to use parental control app frequently.	

*Items were deleted during initial confirmatory factor analysis.
